# Digital Storytelling in Early Childhood: Student Illustrations Shaping Social Interactions

**DOI:** 10.3389/fpsyg.2018.01800

**Published:** 2018-10-10

**Authors:** William Ian O’Byrne, Katherine Houser, Ryan Stone, Mary White

**Affiliations:** College of Charleston, Charleston, SC, United States

**Keywords:** child–computer interactions, human–computer interaction, storytelling, digital storytelling, education, CCI, HCI

## Abstract

This study tests an instructional model designed to empower students in an early childhood classroom as emerging digital storytellers. Educators can use digital storytelling to support students’ learning by encouraging them to organize and express their ideas and knowledge in an individual and meaningful way while developing voice and facility in child–computer interactions. This work also helps develop traditional communication skills, fosters collaboration, and strengthens emergent literacy practices. Students develop enhanced communication skills by learning to organize their ideas, ask questions, express opinions, and construct narratives as they interact with others and computers in the creation of digital stories. The “Emerging Digital Storytellers” instructional model focuses on social-emotional development and finding student voice through writing and digital content construction in the early childhood educational context.

## Introduction

Storytelling has a rich tradition, and it has evolved and expanded to assume a dynamic, contemporary presence across settings and functions. Emergent digital methods are changing the nature of storytelling and opening new possibilities for collaborative approaches. These methods encourage repositioning learners as coproducers of knowledge who partner in the definition of problems, formulation of theories, and the application of solutions in the learning environment. The simplification, interactivity, and affordability of technology has led to a rapid and diverse expansion of participatory storytelling strategies. Digital storytelling has been shown to be a valuable tool to help teachers encourage their students to engage in discussion, participate in instruction, and support the comprehension of content (Kosara and Mackinlay, 2013). This study tests an instructional model designed to empower students in an early childhood classroom as emerging digital storytellers.

The ubiquity of digital texts and tools that can be used to manipulate information and improve student education is increasing every day. Over the past decade, this influx of tools, spaces, and practices (i.e., mobile devices, digital cameras, editing software, authoring tools, and electronic media outlets) have encouraged teachers to utilize many more approaches and technological tools to help students construct their own narratives, and present and share them more effectively (McLellan, 2007). It is hypothesized that digital storytelling can provide many significant benefits to students as they have the opportunity to learn how to create their own digital stories. Students can enhance their knowledge and academic skills as they are asked to research a topic, look for pictures, record their voice and then choose a particular point of view. In this study, our focus was on the use of digital storytelling as a vehicle to help students build capacity for storytelling, engage in literacy practices, and strengthen interactions with others in and out of the classroom. Yet, within these changing dynamics, there are questions about the use of these digital texts and tools in early childhood education (Burnett, 2010; Flewitt et al., 2015). There is a need for increased understanding about the role and place of educational technologies in early childhood educational contexts (Blackwell et al., 2015).

Prior literature has argued that educators should use digital storytelling to support students’ learning by encouraging them to organize and express their ideas and knowledge in an individual and meaningful way (Robin, 2008). We believe work such as this helps students not only develop voice and facility in the child–computer interactions (Iversen and Brodersen, 2008), but it also helps them develop traditional communication skills, fosters collaboration, and strengthens emergent literacy practices. As indicated by Robin (2008), “students who participate in the creation of digital stories may develop enhanced communications skills by learning to organize their ideas, ask questions, express opinions, and construct narratives” (p. 5). The “Emerging Digital Storytellers” instructional model focuses on social-emotional development and finding student voice through writing and digital content construction in the early childhood educational context.

## Theoretical Background

### Storytelling

According to Bruner (1986), “[Narrative] deals in human or human-like intention and action and the vicissitudes and consequences that mark their course. It strives to put its timeless miracles into the particulars of experience and to locate the experience in time and place.” Telling stories allows individuals to narrate our own experiences, and explore or pronounce fundamental elements of our identity. Stories resonate in social settings, and have the potential to pass across backgrounds that often separate us (Alexander and Levine, 2008). Storytelling in the classroom often provides a powerful opportunity to embed elements of narrative, identity, and writing into classroom pedagogy. Stories provide a realistic and authentic opportunity to capture students’ attention and help them listen and learn more actively than other forms of instruction by providing a vehicle to bring facts to life, make the abstract concrete and, through meaning making, make disciplinary literacies more accessible (Isbell et al., 2004).

Young children construct knowledge of their world through the stories they hear and participate with. They interpret and comprehend literary stories by constructing the “world” being described through text (Semino, 2009). When we read or hear stories, different parts of our brain actively track different aspects of the story as if the individual were experiencing the events firsthand (Speer et al., 2009). Rather than mere recipients of the story being told, students become active participants and may help co-construct the narrative (Alonso et al., 2013). According to brain research, storytelling engages areas of the brain related to cognitive control (Lehne et al., 2015), emotion (Hsu et al., 2015), empathy (Brink et al., 2011), and social norms (Berthoz et al., 2002). Stories create an opportunity to help students comprehend and emote while connecting “new knowledge with lived experience and weaving it into existing narratives of meaning” (Rossiter, 2002, p. 1).

Storytelling provides an opportunity to explain and illustrate abstract ideas or concepts in a way that makes them more approachable and accessible. Stories offer a vehicle to bring facts to life, make the abstract concrete and, through meaning making, make disciplinary literacies more accessible (Isbell et al., 2004). Wells (1986) posits that storytelling is a fundamental means of meaning-making as a knowledge construction process. Educators are experts in their field and may be accustomed to using discourse that can intimidate and overload novice learners. Storytelling breaks down the communication barriers between experts and novices and forms an accessible bridge for both to meet intellectually as they collaboratively connect one object to the next (Papert, 2000).

### Digital Storytelling

Creation of digital stories in the classroom is a powerful instructional technique that has the potential to transform learning for students. Digital stories are portable as they are documented and shared via digital texts and tools. This allows the teachers to document the work process and product of the learner, while allowing the students to view the work of others. Products created in digital storytelling transcend traditional classroom assignments as they allow students to explore identity and the meaning of their own experience through multiple avenues (Alonso et al., 2013).

Digital storytelling, like traditional storytelling, focuses on the development of a chosen theme or focal point for the story. In this process, students typically brainstorm, conduct research, write a script, and develop an interesting story. In moving from storytelling to digital storytelling, there is one key difference between digital storytelling and traditional storytelling. Digital storytelling is supported by a variety of digital multimedia tools. Digital storytelling combines a mixture of graphics, text, recorded audio narration, video and music to present information on a specific topic through the use of technology.

In educational settings, different technological tools and programs, such as podcasts, infographics, and other types of presentations, make it easy for instructors to create digital stories (McLellan, 2007; Bower, 2015). Digital stories weave “the art of telling stories with a variety of digital multimedia, such as images, audio, and video” (Robin, 2006, p. 1). Digital stories can be used in the classroom as teasers to pique students’ curiosity about a topic or idea or link prior knowledge to new knowledge (Robin, 2006). Using digital stories to provide a reason why or to introduce a larger topic is one way to use stories that inform and instruct and delve more deeply into an issue (Simmons, 2006). Even with these opportunities to embed digital storytelling in educational settings, there are questions about the role and place of these digital screens and devices in early childhood education. Mentoring students in digital storytelling may seem overwhelming in early childhood educational settings, but the focus should be on student learning objectives, building capacity over time, and supported by plans for mentoring and targeted professional development of teachers. This guidance is most identified in a joint position statement of the National Association for the Education of Young Children and the Fred Rogers Center for Early Learning and Children’s Media at Saint Vincent College, 2012.

Digital storytelling is an especially good technology tool for use in instructional settings as it combines researching, creating, analyzing, and combining visual images with written text (Cherry, 2017). Robin and Pierson (2005) adds to this by indicating that integration of visual elements with written text both enhances and accelerates student comprehension. In addition, digital storytelling has a variety of applications in the classroom, including the telling of personal stories, narrating past events, or as a means to teach on a particular topic (Jakes, 2006). Most of the work on digital storytelling began in 1990 as Joe Lambert developed digital storytelling in the virtual world as the cofounder of the Center for digital storytelling (CDS). Since that point, the CDS has been influential in developing and disseminating the Seven Elements of Digital Storytelling (see **Table 1**), which aids teachers in creating digital stories with their students (Robin, 2008). Creating digital stories in education brings with it a number of different variables that impact instruction and student interactions.

**Table 1:** Seven elements of digital storytelling.

(1) **Point of view.** Told for a specific purpose or to make a point for a given audience.
(2) **A dramatic question.** Gives a reason for the audience to stay interested; answered question by the end of the story.
(3) **Emotional content.** Images, tone and effects connects the story to the audience.
(4) **The gift of your voice.** Personalizes the story for the audience to help them to understand the context.
(5) **The power of soundtrack.** Music or other sound that supports the storyline and conveys emotion.
(6) **Economy.** Uses just the necessary elements to tell the story.
(7) **Pacing.** Controls the story; and how slowly or quickly it unfolds.

Digital storytelling has been shown to be a powerful collaboration tool that teachers have used to support student collaboration and communication. The tools and practices included in digital storytelling have been useful as teachers encourage students to prepare their own stories for their peers and connect with others in and out of school. Teachers can create digital stories as inspired from content, or have students express mastery of the content in digital stories. The most powerful example of the use of digital storytelling may be instances where students are asked to create their own narratives either individually, or as members of a small group (Sadik, 2008). In this study, we worked with students in an early childhood environment to have students work individually as they expressed and storyboarded their stories, and then were mentored into the digital storyboarding process by classroom teachers.

### Child–Computer Interactions

Child–computer interaction (CCI) is an evolving area of research that focuses on the reciprocal actions between children and the Internet and other communication technologies (Read and Markopoulos, 2013). CCI is a research discipline within human-computer interaction (HCI) which is multidisciplinary in nature and informed by work in a variety of fields (e.g., educational psychology, developmental psychology, learning sciences, computer science, game design). These two fields (i.e., CCI and HCI) are emerging, and as such require insight from a variety of fields, yet also need opportunities to remain flexible and account for changes in technologies, and our understanding of these elements (Read and Bekker, 2011).

Read and Bekker (2011) define CCI as the “study of the Activities, Behaviors, Concerns, and Abilities of Children as they interact with computer technologies, often with the intervention of others (mainly adults) in situations that they partially (but generally do not fully) control and regulate.” Children in CCI are identified as individuals between the ages of 5 and 12, but increasingly this lens has included toddlers and adolescents in this focus. As technology becomes more ubiquitous in society, there are questions about the growing need or purpose for children to use ICTs during critical developmental periods (Plowman et al., 2010).

Children now grow up surrounded by a plethora of screens that may be concerning to adults (Pollock et al., 2010), yet they also may be a hallmark of our networked society (Plowman and Stephen, 2003). This access and abundance of screens, and the questions or concerns about CCI may be partially dependent on a variety of factors, but children in the most developed countries are some of the most frequent users and consumers caught up in the challenges and opportunities present in CCI. This study examines the role of CCI within a population of 4, 5, and 6 year old students in an early childhood educational setting.

Given the emerging challenges and opportunities that exist in CCI, and the potential for applications of these technologies as an educational tool, there is an urgent need to explore how current and future HCI will impact learners (Read and Markopoulos, 2013). Educators are making assumptions that developers, researchers, and organizations are delivering technologies that will improve student learning outcomes (Hess and Saxberg, 2013) without negatively impacting individuals (Punchoojit and Hongwarittorrn, 2015). These new developments and technologies also need to be matched to best practices and contemporary paradigms in educational psychology to best scaffold learners (Gilutz, 2009).

Classrooms, especially early childhood educational environments, can provide challenging environments for testing and evaluation of these digital texts and tools (Dhir and Alsumait, 2013; Read and Markopoulos, 2013). More to the point, the American Academy of Pediatrics (2016) has indicated that “screen time” for entertainment for children should be modified for specific age brackets of children. The AAP suggests avoiding use of screen media other than video-chatting (Council on Communications and Media, 2016a) for children younger than 18 months. The guidelines suggest children 18–24 months of age can view digital media of high-quality programming, whereas children aged 2 to 5 should be limited to 1 h per day of high-quality programming. Children aged 6 and older should have consistent limits placed on the time and types of media consumed. In all cases, the AAP (Council on Communications and Media, 2016b) In all cases, the AAP recommends co-viewing of content and subsequent discussions between children and parents or guardians to help children understand what they are viewing (Council on Communications and Media, 2016a,b). The AAP also recommends developing these guidelines with children to make sure media does not take the place of adequate sleep, physical activity and other behaviors essential to health. Research suggests that less than half of the time spent in front of screens by children aged 2–10 is spent consuming content that is educational in nature (Rideout, 2014). Thus, research such as the one presented in this study is needed as we strive to not only evaluate new HCI and CCI interactions, but also test the techniques used to make these connections.

### Computer Supported Knowledge Construction

Numerous skills and strategies are needed in both the procedural and strategic use of digital texts and tools in storytelling. The knowledge, skills, and dispositions involved in this instructional model are informed by previous research in writing and storytelling instruction (Collins and Gentner, 1980; Flower and Hayes, 1980; Hayes and Flower, 1986; Scardamalia and Bereiter, 1994) and envisioned as a combination of skills students use to construct stories and digital content. The five skills involved are *planning, generating, organizing, composing*, and *revising*. Planning is defined as a student creating internal and external representations of the content they intend to build and ensuring that it is logically appropriate for the task (Flower and Hayes, 1980). These representations may include paper sketches, graphic organizers, or original designs of future works planned. Generating is defined as the process in which a student creates or translates initial elements of the digital product based on their memory and organizers (Collins and Gentner, 1980; Hayes and Flower, 1986).

These initial drafts and modeling activities act as elements of the work completed to allow the student to begin reviewing and organizing materials. Organizing is defined as the process in which a student creates or manipulates the hierarchical or relational structure of their work product (Flower and Hayes, 1980). In this process students maneuver content and categories of content to ensure they meet the goals of the inquiry and purpose of the content. Additionally, as students organize, they may attend to aesthetic decisions about the presentation and ordering of elements of the content (Hayes and Flower, 1986). Composing is defined as the process in which a student constructs the online content while weaving elements from the previous three phases into a cohesive composition that is representative of the goals of the inquiry process. Revising is defined as the process in which a student dedicates time to systematically review and examine with the intent of improving the overall work product (Hayes and Flower, 1980). The process of reviewing and revising may occur across all stages of the model, however, this final step is one in which students consciously examine and evaluate constructed content before finishing the work process.

Embedded within each one of these five skills is a recursive, metacognitive review process in which students retrospectively considers their ideas, evaluate this work in relation to the task or purpose, and possibly share with others to obtain another perspective on their work. Much of this review process is informed by the complex pattern of goal setting, problem solving, and reflection known as “knowledge transformation” (Scardamalia and Bereiter, 2006). Embedded in this process is an examination of the differences between “knowledge-telling” and “knowledge-transformation” strategies (Scardamalia and Bereiter, 2006). Knowledge-telling strategies were defined as the retrieval from long-term memory of ideas related to a rhetorical goal and their resultant transference into text (Scardamalia and Bereiter, 2006). Knowledge-transformation strategies were defined as those ideas that were transformed in an effort to resolve a conflict between the original ideas and the intended rhetorical goal (Scardamalia and Bereiter, 2006). This review process has the potential to result in the generation of new knowledge and a deeper understanding of the student’s content knowledge (Collins et al., 1991; Scardamalia and Bereiter, 2006).

## The Aim of the Present Study

This study focuses on how the children in an early childhood classroom establish their voice through storytelling. More specifically, the study examines the mentoring and modeling of storytelling and digital storytelling within a mixed-age classroom (PreK and K) by two teachers (White and Stone). We examine the mentoring and modeling process as the classroom teachers explore the use of digital technologies and their effects on the child’s motivation to write, create, and share their stories within the classroom community. As such, this study focuses on two related research questions.

• What are the themes and patterns that exist in the process of story development and ways in which early childhood children share personal stories in an early childhood educational environment?
• What is the role of child–computer interactions and educational technologies in shaping content and social interactions as students engage in the process of story development in an early childhood environment?

To answer these questions, our research examined the process of story development through the intersection of CCI within an early childhood classroom environment. As researchers and educators, we were concerned with the social-emotional development of the child when working or collaborating with their peers, the process of story development and layers of story involved in the final product, and developmentally appropriate ways to engage children with CCI as they develop a sense of self through the sharing of stories. We focused on the mentoring and modeling of storytelling as defined by the two classroom teachers (White and Stone). We also focused on the mentoring of students as they are provided exposure to potential future uses of CCI and HCI. This qualitative, participatory action research (Whyte, 1991; Merriam, 1998) investigated the use of an instructional approach focused on storytelling and digital storytelling in an early childhood classroom to explore the impact on the learner’s connection with computers, content, and classmates.

## Materials and Methods

This study is part of a larger research project in which we are focusing on the child’s motivation for writing, the impact of CCI, and the implications for future teacher practices to see what the results may reveal. This work all centers on the development and use of an “Emerging Digital Storytellers” instructional model in which we focus on social-emotional development and finding student voice through writing and digital content construction. This model also builds opportunities to mentor students in positive CCI and HCI, while also working collaboratively and cooperatively with peers. As informed by the earlier section on CCI, we believe that it is important to only expose these young children to digital texts and tools when it is a meaningful extension of the content and curriculum. We also believe in safely studying our own practice, and being considerate of the risks and opportunities present in CCI exposure at this age. That is the motivation for this area of inquiry.

### Participants and Procedures

This study was conducted with a convenience sample from a mixed age class of 4, 5, and 6-year-old students in an early childhood classroom (*N* = 25). This classroom is part of a larger early childhood development center that is facilitated and associated with a small, public university in the southeast of the United States. The student population represents families associated with the college or local community. As a demonstration program, the school structures class groups to support appropriate diversity that reflects the surrounding community, gender distribution, and accommodation for children with special needs to model best practices for pre-service teachers.

The early childhood development center focuses pedagogy and student engagement on a play-based, emergent curriculum. The class is taught by the second and third authors (Stone and White). The first author (O’Byrne) is an assistant professor of literacy education at the college. He served as a participant researcher (Angrosino and Mays de Pérez, 2000) in the role of an objective observant (Punch, 1998) in the project. Instruction was also provided by two teaching assistants in the classroom that were not included in data collection or analysis for this study. Their instruction in the classroom was overseen by the two classroom teachers at all times. The researchers obtained written informed consent from the parents and guardians of participants in this study.

To address the research questions we conducted a two-phase analysis of qualitative data to inductively analyze (Patton, 2002) and ultimately develop themes (Merriam, 1998) from the data. Data consisted of three different sources to allow for triangulation of findings (Denzin, 2012): (a) student work product (i.e., sketches, illustrations), (b) video recorded observations of students in the classroom, and (c) researcher notes. Phase one of the analysis consisted of instruction and regular meetings of the research team to understand initial learner dispositions and possible changes in these dispositions during, and after exposure to elements of storytelling, animation, and digital storytelling. Phase two had the researchers go back to the dataset and identified themes to find commonalities and trends in the data on a more deeper level.

### Instructional Model

Within the Emerging Digital Storytellers instructional model, there is very little direct instruction by the teacher and limited use of graphic organizers. There is, however, a great deal of scaffolding and modeling by classroom instructors, and the use of student work product as a means to motivate and illustrate story elements to other students. A typical day in this classroom includes time set aside each day for this modified version of “Story Workshop” (Shiflett, 1973; Schultz, 1990) which allows students a formal, guided setting where teachers provide prompts such as “What is your plan?” and “Tell me about your story.” Stories are written in an open-ended context in which they can be written in one setting or over several days. They are usually shared with teachers, peers, and parents.

Student stories usually consist of hand-drawn images on a variety of paper and journaling notebooks. In addition to the stories regularly created and shared, each student is given an opportunity to have a digital version of their story created by one of the classroom teachers (Stone). The process for creating this digital version is explained below, as well as more information about the writing and storytelling process. As such, in this study, the students are mentored in two examples of storytelling. One of which is digital and one that could be considered to be traditional. Each of these is introduced and viewed by the teachers and students as being equivalent, and both incorporate the students writing, drawing, and voice in the final work product. Videos of all aspects of this instructional model are available in the **Supplemental Materials** section of this publication.

#### Mentoring in Traditional Storytelling

As co-producers of knowledge, children in this model are encouraged to share their stories in an emergent process that is wholly owned by the student. There is no specific, direct instruction of storytelling as we are striving to capture the normal acts of storytelling that are shared during time in the model, or observed during play in work centers and outside. Stories are shared without any visuals at all and rely completely on the audience to use their imagination. In some instances, visuals can be used to enhance understanding and provide cues to drive home the meaning of the story. Visuals can take the form of photographs, drawings, or in the case of this study, animations. In our instructional model, the creation of animations, or digital versions of student stories is a natural connection as CCI is used to bring the story to life, and capture a moment in the lives of students. The digital version of the story provides an archive or assessment of the student and their storytelling, and literacy practices recorded in time. To capture these stories, students first draw out, or storyboard, their stories and relate them verbally to the classroom instructors. They often use their art, voices, and bodies to enact the story or develop drawings. For the purposes of this study, student art, video recordings of the class, and researcher notes were collected and analyzed to understand these themes.

In this model, instruction is focused on the child as the expert in his or her reality. As such, students are provided time to “free write” where they may engage in initial conversations with peers or adults about what they are going to write about. In small groups of 6 children, each student illustrates their story in their journal and when finished the teacher makes a “teacher note” where they record, verbatim, the story told by the child. The child’s image may look like scribbles, basic shapes, people, objects, layered drawing, random letters, or any number of other ways students choose to represent their thoughts and stories using pencil and paper. Next, students may be prompted to write or sound out words for their story based on their readiness and developmentally appropriate practice. It is common for stories to change during this time where the student is influenced, directly or indirectly, by peer comments, stories, or questions. For example, a student may be storytelling about ninja turtles when their peer shares their story of the two of them riding bikes. The story may change as the turtles are riding bikes, or friends appear in the turtle story as a result.

Embedded in this process is also a continual process of creation, review, feedback, and recreation. In terms of fully understanding the complexity of this metacognitive review process, it is important also to understand how the knowledge-telling and knowledge-transformation strategies espoused by Bereiter and Scardamalia have been revised. Galbraith (1999) identified “knowledge-constituting” as involving a “dialectic” between dispositional aspects of students as they attempted to make sense of their thinking as they constructed knowledge (Galbraith, 1999). This dialectic involves the student engaging in the processes detailed by Scardamalia and Bereiter (2006) but modifying it with each additional element of text that was constructed (Galbraith, 1999). This informs the review process by involving a cycle in which the students construct knowledge in the form of text and then considers if this idea is satisfactory or not (Galbraith, 1999).

This knowledge construction process grows more complex as students must consider the effect of multimodal content such as images, video, and audio and the effect this has on their work product (Duncum, 2004; Sheppard, 2009). Students may consider visual aesthetics, elements of graphic design, and semiotic elements that may affect how the audience perceives their work (Serafini, 2011). This instructional model builds on this by providing students with modeling, and a possible connection between storytelling and digital storytelling.

#### Mentoring in Digital Storytelling

We utilized a variety of digital texts, tools, and software to digitize the stories created by participants. This includes the use of a snowball microphone or camera to capture audio and a scanner or digital camera to capture pictures. Additionally, Adobe Photoshop and Premiere was used to animate student stories while keeping their voices in the final work product. Finished products were shared with students via animations and printed stories with CD-ROMs at the classroom listening center. The goal is for student voices to be captured and celebrated where peers, teachers, and families become familiar with student stories and share or retell them.

This use of technology in storytelling allows students to engage their peers in the telling of a story (Lisenbee and Ford, 2017). The creation of an animation allows for the documentation of the student at one point in time, their images, and their voice. The use of technology within this classroom is seen as a means to capture or document student work. By animating a child’s story, teachers provide a greater level of engagement and understanding by visually representing the action through the movement of the child’s drawings, adding sound effects, and having the child narrate.

This move to digitizing, or creating animated versions of student stories began organically in this class. Initially seen as an opportunity to motivate students to spend more time on their stories, story animations authentically used technology as a tool to capture and bring to life the child’s illustrations utilizing their voice and images to tell their story. This model began the previous school year when a child wrote a simple three-page story at the art center during center time. One of the teachers (Stone) brought over 10 years of movie editing experience into the interaction by deciding to scan the story and record the students voice on the computer. “I’ve seen how people use animations and short videos on social media and this was the next thing I wanted to try” (R. Stone, personal communication, April 4, 2018). Using editing software, he cut the images out using Adobe Photoshop, made them move in Adobe Premiere, added simple sound effects (i.e., footsteps, cars driving, babies crying). The final animation was shared first individually with the student, and then her family, and finally the classmates. The classroom teachers noticed the impact of this CCI on the social connections and learning in and out of the classroom. The ability of the animation to document a point in time in the development of the learner, as well as being portable enough to share out to others in made it a valuable form of currency in the learning pathway of individual students.

As Reiber (1990) describes, animation is often used for one of three functions: attention-gaining, presentation, or practice. The moving image and sound effects capture student attention, engage students, and motivate reluctant storyteller. In this process, the teacher models the use of technology as an innovative way for students to reach their audience. After animating four or five stories, the stories are added to a listening center in the classroom by burning the audio to a CD-ROM and printing each students’ story and putting them together in a presentation book. Students enjoy listening to their peer’s stories and quickly memorize each, as they would a story that has been read to them multiple times. The animations are used to document student work and share with parents as a memory or keepsake (Read and Markopoulos, 2013).

### Data Collection and Analysis

As previously indicated, data were collected and analyzed over two phases. Phase one included data collection over the course of 4 months in which participants regularly constructed traditional stories, and one by one, students had their stories translated into digital versions. Phase two of the analysis was conducted after the completion of instruction and collection of all data.

#### Analysis Techniques

Qualitative data on participants were collected and analyzed to answer the research questions. Critical to the process of qualitative data analysis is ensuring that data collection, management, and analysis operate in concert. Therefore, consistent with qualitative research guidelines, these three processes occurred simultaneously throughout the duration of the first phase of the study (Creswell, 2007). This process insured the recursive nature of data collection and analysis necessary in naturalistic qualitative inquiry (Patton, 2002). Analyses of patterns and themes in qualitative data allowed the dynamics of change to be more evident and permitted us to better understand how participants’ comprehension and levels of CCI changed over the course of the study. Data consisted of recordings of participant work sessions and presentations of their work, as well as completed work product, and researcher field notes. Data were analyzed in a multi-step process to recognize patterns (Patton, 2002) and to develop themes (Merriam, 1998).

From the initial phase of data collection and analysis, we began to identify emerging patterns that enabled us to ask additional questions to promote greater exploration. We used these patterns as a guide to pursuing subsequent data collection (Denzin and Lincoln, 2005). We started our analysis with open coding of the videos of student work. The codes are “tags or labels for assigning units of meaning to the descriptive or inferential information compiled during a study” (Miles and Huberman, 1994, p. 56). Once a week, the research group (i.e., O’Byrne, Stone, and White) would discuss the themes, categories, and codes identified in the analysis. In addition to these weekly meetings, the research group was joined by one of the teaching assistants for review during each pass through the data in order to check this work against the research question (Maxwell, 2012).

## Results

As stated earlier, the analysis of data was conducted in two phases. This provided the research team with two stages of themes, categories, and codes from the data with informed our research questions.

### Stage One Themes

The first stage of analysis included successive passes through the dataset to allow for data reduction and data synthesis. Weekly research meetings served as debriefing sessions to allow us to compare codes and themes while examining their relationship to the research purposes (Thomas, 2006). In this process, the research group identified a series of open codes, which were then used to continue analysis through continued passes through the data. This process included reading closely across the video data, taking notes, and creating preliminary inductive codes. A series of three themes was developed (Patton, 2002) from the original set of open codes:

1. Storytelling: Participants utilized scaffolding and mentorship in the instructional model in a variety of ways that impacted how they profited from the experience.
2. Digital storytelling: Participants valued and regularly edited their work using comments from teachers and peers in order to have their work effectively translated into a digital format.
3. CCI as currency in the classroom: Educators effectively leveraged the instructional model as an opportunity to provide exposure to creative and expressive forms of CCI use in and out of the classroom.

While useful, the Stage 1 analysis was critical to developing the initial phase for the more in-depth Stage 2 analysis of data.

### Stage Two Themes

Recursive, analytic inductive methods (Angrosino and Mays de Pérez, 2000; Bogdan and Biklen, 2003) were used to make additional passes through classroom observational video, field notes from classroom observations, and student work products. During the second stage of analysis, several themes and their associated dimensions emerged. These patterns were further distilled as successive passes through the data were made to refine the initial structure. This analysis included an iterative process that involved reorganizing the data and reworking the groupings so that the category structures and themes defined items adequately and represented primary trends in the data.

After the initial coding, constant comparative methods (Glaser and Strauss, 2017) were used across all codes to collapse the preliminary codes into specific categories. Once categories were created, a rigorous content analysis (Mayring, 2000) was used to organize the field and to identify themes that were interpretative of the research questions. Illustrative case studies (Davey, 1991) were constructed using information from students in the study. These studies were developed with themes from the second level of analysis to make them easier to understand. The case studies presented leant insight into “important variations” (Davey, 1991) in the data and the knowledge, skills, and dispositions of the students. Each case study provides insight into the four levels of student as identified in the data in this study. The following stages were created to assist classroom teachers in identifying where students were in their storytelling ability, and to use for training purposes with assistant teachers. These stages reflect the child’s *storytelling* ability, not their ability to represent their story through pictures or words.

#### Stage 1: Pre-storytelling

Students at this initial stage have difficulty coming up with a story without some form of adult instruction or guidance. They often draw or scribble, then make up a story when prompted. There is no planning or forethought and the story may change multiple times based on when you ask for retell. The child may tell the same simple story every day (repetitive) over many weeks or months.

There is also little to know understanding of how to prepare a story for later use in digital storytelling. The student in this stage is not only building their literacy skills, but also their storytelling ability. The student in this stage understands that student stories are focused on in the digital stories, but cannot figure out the process involved, or how to modify their work to assist in this interaction with the computer.

**FIGURE 1:**
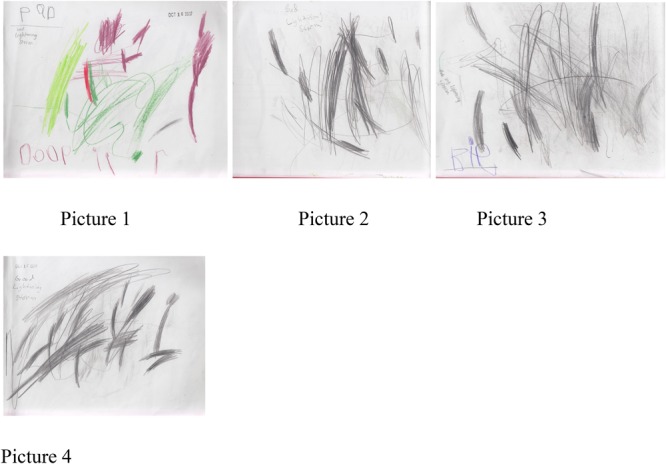
Pictures 1–4 detailing a story from Lewis, a 4-year-old student.

Lewis, a 4-year-old male student wrote, “A bad lightning storm,” (picture 1, 10/16/17), “Bad lightning storm, good lightning storm,” (pictures 2 and 3), and “The big lightning storm,” (picture 4). See **Figure 1** for pictures 1–4. This student often draws an image first, then will say a similar story several days in a row or will revert to the same story frequently. A recent hurricanemade landfall in the local area and this impacted the lives of all students, which became a common thread in many student stories that semester. Students in Lewis’s group also wrote about their homes being flooded or having to leave and stay with relatives during the weeks that followed. Researcher notes indicate that Lewis preferred to write about the storm when other ideas did not present themselves. “When he doesn’t want to write he’ll write about a storm.”

#### Stage 2: Developing Storyteller

Students at this stage are now able to stick to a familiar story script where characters and actions follow a particular set of rules. The characters have particular roles (i.e., good guy, bad guy) or series of actions (the prince married the princess) and the child adheres solely to these designated roles. They may cite factual information from non-fiction texts of particular animals or other areas of interest, however, cannot use those facts to create a story. Storytelling at this stage often looks repetitive in nature and where the child sticks to what is known to be true (familiar TV show or movie, fact book, etc.).

Students at this stage also begin to show awareness of CCI, and are motivated by the opportunity to have their story digitized and shared with others. As they are beginning to understand the process of storytelling, they are not at all proficient in CCI, or ways to modify their story to allow for future digital storytelling efforts. Students at this stage are excited and perhaps motivated by the opportunity to have their story digitized by the classroom teacher, but their is little to no awareness or understanding of how their work plays a role.

Norman, a 4-year-old male student, is obsessed with Transformers. His stories all reflect his knowledge and understanding of either a particular movie or show. Each story has the same characters playing their designated roles, Optimus Prime is the good guy, Megatron the bad guy. These characters fight and the good guys always win. Researcher notes capture a conversation Norman had with a teaching assistant as he creates and draws his story.

Teacher: “Will you tell me what’s going on in your story?”
Norman: “Um, MassPrime was fighting a lot of bad guys and then he punched Megatron (motions with right arm) and Megatron, MassMegatron, Megatron in the face.”
Teacher: “OK”
Norman: “The End. Then he gets allll trophies.”
Teacher: “OOOOK”
Teacher: “What’s your hand?”
Norman: “Uh, it’s covered. I have to get one more trophy. (chooses another pencil and starts to color). There you go.”

Teacher writes on student’s story: “Megatron punched Masstron in the face. He got a trophy!”

As a developing storyteller, Norman has a set script that he is familiar with and comfortable retelling. This can be observed in his play at the water table, in blocks, and on the playground where any inanimate object represents Optimus or Megatron and they act out this familiar story.

**FIGURE 2:**
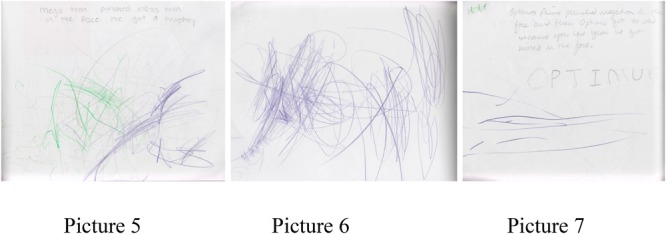
Pictures 5–7 detailing a story from Norman, a 4-year-old student.

“Optimus Prime punched Megatron in the face and then Optimus got so sad because after last year he got hitted in the face” (pictures 5–7). See **Figure 2** for pictures 5–7.

#### Stage 3: Emerging Storyteller

At this stage, students begin to create new and unique stories based on familiar information. They may use familiar characters in a new context or situation or draw simple stories of something that happened to them personally (family stories). A big part of this stage is the creativity that emerges as students feel comfortable and confident with the repertoire of stories and events that have been either experienced or shared with them. Their stories grow in length and include more action and descriptive words. There may be a simple sequence of events that includes a basic beginning, middle, and end.

Students at this stage are also a bit more advanced in their understanding of CCI, and how to prepare a story to ultimately be scanned and turned into a digital story. This means that a student may understand that it is challenging to scan a story that multiple layers of images that have been drawn or scribbled over. The students also understands the need to storyboard out their narrative, and include individual pictures or graphics that are easy to scan and separate later in the process on the computer. Put simply, they prepare their work in a way that makes the teacher’s use of the computer in editing and animation much easier. This may be learned by watching or reviewing the work process or product of students in the class.

**FIGURE 3:**
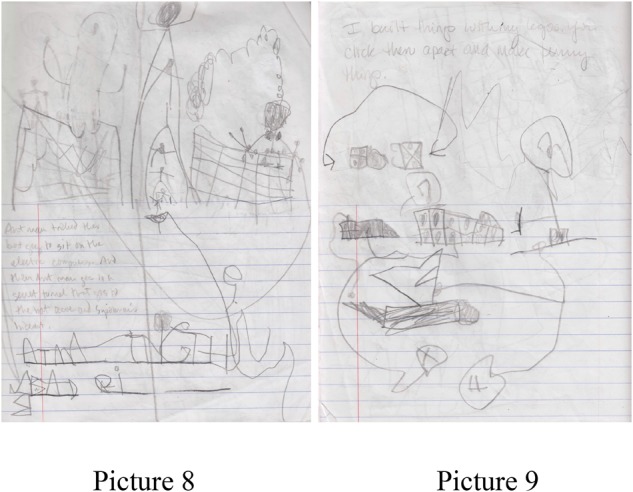
Pictures 8 and 9 detailing a story from Orlando, a 6-year-old student.

A 6-year-old male student, Orlando enjoys playing with Legos, Mixels, and is familiar with superhero stories. His stories reflect a combination of a variety of characters interacting in new and creative ways. He has moved away from a script and can write both fiction and personal narratives, expressing what is familiar to him: “Antman tricked the bad guy to sit on the electric computers and then Antman goes to a secret tunnel that goes to the batcave and Superman’s hideout” (picture 8). On other days this student can recount a personal event: “I built things with my legos. You click them apart and make funny things” (picture 9). He is able to tell the difference between real and make believe. See **Figure 3** for pictures 8 and 9.

**FIGURE 4:**
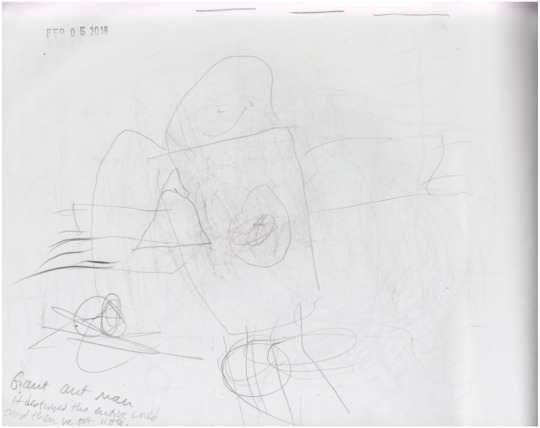
Pictures 10 detailing a story from Orlando, a 6-year-old student.

In small groups, students may have an influence on the work of their peers. On this particular day, Orlando’s peer was also writing about Antman (picture 10). See **Figure 4** for picture 10.

“Giant ant man. It destroyed the whole world and then he got little.” Orlando would lean over and ask questions about his peer’s story and his peer would do the same. Often times peers will add their friends or will ask to be a part of their friend’s story, “Can I be the baby horse?”

#### Stage 4: Early Storyteller

Students are now able to work independently to create a story of their choice. There is little guidance or instruction and the student often has a plan before beginning their storytelling. The child can differentiate between fiction and non-fiction and may choose to write one type of text or another. For example, writing about different types of birds over several days by describing each. Students begin to sound out text on their own, and may choose to piece the story together over several days (beginning of story mapping). They are comfortable using transition words when retelling and these may be seen in their writing.

Students at this stage are advanced in their understanding of process and how their work product needs to be constituted to make it easier to scan and turn into a digital story. A student at this stage will fully storyboard out their story and often have individual pages contain individual images as part of their story. Students at this stage will often include more illustrations and pages to convey more information in the story, as opposed to layering action on top of earlier images. As opposed to students in the Emerging Storyteller stage, students in this stage will be the ones that tell other students how to prepare their work to make it easiest for the later use of computers. Students in this stage will tell other students, “You can’t draw your story like that because Mr. Ryan can’t scan it later.” This understanding of work process and how it informs the desired product is an advanced interaction between the child and computer.

**FIGURE 5:**
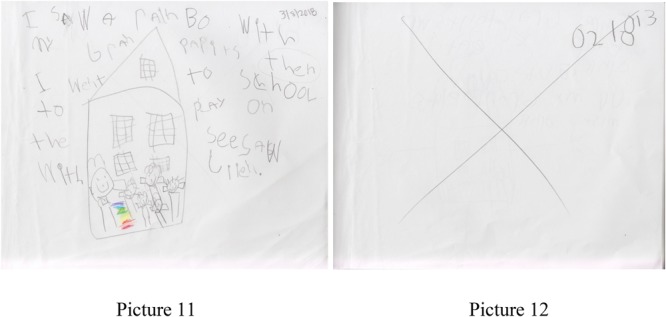
Pictures 11 and 12 detailing a story from Sally, a 5-year-old student.

Sally, a 5-year-old female student, usually writes in coordination with several of her friends. They write on the same themes and consult with the teachers as they draw. An example of this would be a question Sally asks as she draws a version of Thomas, another student, “What color shirt do you want me to draw for you?” In another example, Sally told the classroom teacher “I’m going to write about my grandparents.” She had a plan for her story when she arrived at school, and immediately sat down to draw and write when she entered the classroom. She crafted her story with some input from Rose, a 6-years-old peer, to help her spell rainbow and parents. She reviewed her story by reading, “I saw a rainbow with my grandparents then I went to school to play on the seesaw with Jiiel” to her tablemates (picture 11). See **Figure 5** for picture 11.

Sally also developed her own advanced form of notation in storytelling as a way to convey information to herself, peers, and ultimately the classroom teachers. As an example, Sally uses the + sign for transitions. In picture 11, she explains, “Thomas and I were puppies and Mr. Ryan was our daddy,” so the use of “then” was a new use for her. In another example, in picture 12, she drew a picture and a related story by exclaiming that “Now I will write about a sleepover with Thomas.” After completing the story, she dated the page and put a big X across the page and said, “I don’t want to write that today.” See **Figure 5** for pictures 11 and 12. This use of notation to serve as a peritextual feature to the story was developed on her own and helped guide the work process in the class. Furthermore, the ability to create a story, decide to focus on one story for the animation process, and once again use notation to guide the later interactions with the computer is an accelerated awareness of the role the digital translation will play in this work.

**FIGURE 6:**
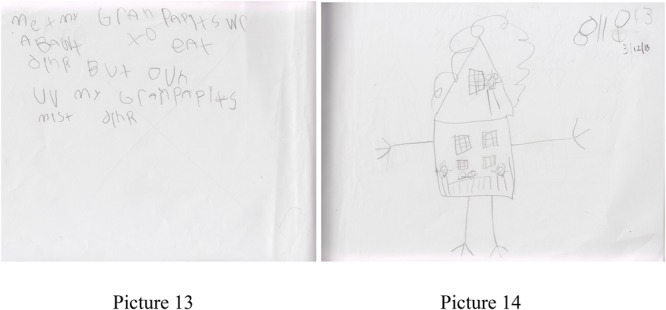
Pictures 13 and 14 detailing a story from Sally, a 5-year-old student.

After an absence of several days, Sally checked her previous story and then added to the story by writing “Me and my grandparents were about to eat dinner but one of my grandparents missed dinner” (pictures 13, 14). See **Figure 6** for pictures 13 and 14.

## Discussion

Digital storytelling provides an authentic opportunity to develop the different types of literacy that students will need in the 21st century. This instruction and knowledge construction process expands to improve various literacy skills and competencies ranging from information literacies (Robin, 2006), media literacy (Jakes, 2006), and visual literacies (Simmons, 2006). The challenge is that in order to make these digital practices come alive in the classroom, there is a need to start building these skillsets at an early age. Furthermore, there is a need to build authentic awareness about the interactions learners may have with computers now and in their futures. There is little reassurance that young children are receiving positive, or informed exposure to computer interactions at home, or in school (Paciga and Donohue, 2017).

In this study, early childhood students are creating their narratives, and being guided by educators as they develop digital versions of these stories. It is hypothesized that we are identifying new possibilities for sharing and creating through the use of digital tools in early childhood. These skills and practices will prove invaluable as students develop the skills and competencies needed to communicate now and in the future. In the future, these practices may help to increase students’ motivation to learn and increase their desire to complete their digital stories as they utilize a variety of multimodal tools while working with text, still images, audio, web publishing, scanners, digital still cameras, video cameras, music, and sound effects.

In essence, students are being mentored into the storytelling process, and the digital storytelling processes by masters in pedagogy in these areas. Furthermore, they are also being mentored into informed CCI as these teachers explore their own understandings of the role and purpose of HCI. For example, we see Lewis being mentored in storytelling by the teachers and peers as he was inspired to write or mashup new content by the ideas and stories of the individuals around him. Norman was mentored in storytelling and digital storytelling as he received feedback from teachers and peers on adding more details to his sketches and organizing this into storyboarding cells to make it easier for the teacher to animate. Orlando showed a skilled understanding of storytelling and digital storytelling, and helped to mentor other students as he guided them by sharing his stories and sketches, and giving feedback on the work of others. As the “early storyteller” in the group, Sally not only helped to mentor and inspire her peers in this work, but also helped the classroom teachers modify the process and learning task by creating shorthand comments on her sketches that helped the teacher understand the vision she had for the animation. The classroom teachers were also a part of this process as they mentored each other in storytelling and digital alternatives by collaboratively teaching the class, and reflecting on these experiences with the researcher.

To make this model a success in the classroom, there is a need for targeted professional development as well as time and latitude for teachers to collaboratively try out this work with students. Targeted professional development is needed to help educators build the skillsets needed to understand and use these tools, but also the willingness to play and explore uses on their own. Mentorship, and apprenticeship models are also an important aspect of this work as the culture of the classroom and early childhood center created a space in which students and teachers were given permission to learn, explore, and play. As detailed earlier this early childhood center focuses pedagogy and student engagement on a play-based, emergent curriculum. The culture established in this environment, and the trust or respect given to the learner and time spent on task may have been a large part of the success in this model. There is also need for research and investigation of the use of these technological tools and practices in real-world childhood educational contexts (Blackwell et al., 2015). A failure to provide early childhood educators with mentoring or professional development in this work adds to a digital divide in which educators and students are not provided equitable access to effective instruction on using technology for authentic purposes and personal empowerment (International Literacy Association, 2017).

There are several limitations to this work as we need to account for the population in which our study is situated. We also need to further explore the role of collaborative and cooperative learning and revision in this work. There is also a desire to understand the role of these stories and their digital counterparts as currency in and out of the classroom. Future research will focus on the importance of risk-taking in sharing one’s narratives with others, while at the same time building learner resiliency and engagement as writers in a community. Further analysis and reporting will unpack these limitations and questions, but these results provide insight into the role of identity and voice in storytelling, and the effect to which animations or visuals help to motivate the writing process.

## Conclusion

Digital storytelling, as mediated by child-computer interactions is a powerful and beneficial pedagogical opportunity to teach and empower students. More specifically, in an early childhood educational setting, these elements have the potential to help develop academic skills and motivation in students. Digital stories are portable as they are documented and shared via digital texts and tools. This allows the teachers to document the work process and product of the learner, while allowing the students to view the work of others. Products created in digital storytelling transcend traditional classroom assignments as they allow students to explore identity and the meaning of their own experience through multiple avenues.

This research expands knowledge in the field as it applies to how the child establishes their voice through storytelling. This medium and the associated tools incorporate higher order thinking skills while also strengthening social connections in and out of the classroom. In particular, this provides more insight into CCI, and the use of digital technologies and their effects on the child’s motivation to write, create, and share their stories within the classroom community.

## Author Contributions

RS and MW taught the study context. KH is the director of the early childhood development center.WOis an assistant professor of literacy education at the college, served as a participant observer in the research, main point of contact, and PI on this study. RS, MW, KH, and WO all contributed to the design of the study, data collection, analysis, and writing of this article.

**STORY SAMPLES 1 |**https://www.youtube.com/watch?v=TWVj6gE2jV0

**STORY SAMPLES 2 |**https://www.youtube.com/watch?v=px9bxjRPKRg

**HOW TO VIDEO |**https://www.youtube.com/watch?v=8Eo2wShgxfY

**FINISHED PRODUCT |**https://www.youtube.com/watch?v=2lVzdIWDIhM

## Conflict of Interest Statement

The authors declare that the research was conducted in the absence of any commercial or financial relationships that could be construed as a potential conflict of interest.

## Supplementary Material

The Supplementary Material for this article can be found online at:

**STORY SAMPLES 1 |** https://www.youtube.com/watch?v=TWVj6gE2jV0

**STORY SAMPLES 2 |** https://www.youtube.com/watch?v=px9bxjRPKRg

**HOW TO VIDEO |** https://www.youtube.com/watch?v=8Eo2wShgxfY

**FINISHED PRODUCT |** https://www.youtube.com/watch?v=2lVzdIWDIhM
