# Corrigendum: Digital Storytelling in Early Childhood: Student Illustrations Shaping Social Interactions

**DOI:** 10.3389/fpsyg.2018.02749

**Published:** 2019-01-16

**Authors:** William Ian O'Byrne, Katherine Houser, Ryan Stone, Mary White

**Affiliations:** College of Charleston, Charleston, SC, United States

**Keywords:** child–computer interactions, human–computer interaction, storytelling, digital storytelling, education, CCI, HCI

Katherine Houser was not included as an author in the published article. The original article has been updated and the new Author Contributions statement is: “RS and MW taught the study context. KH is the director of the early childhood development center. WO is an assistant professor of literacy education at the college, served as a participant observer in the research, main point of contact, and PI on this study. RS, MW, KH, and WO all contributed to the design of the study, data collection, analysis, and writing of this article.”

Additionally, in the original article, there was an error. Incorrect demographics were included for the center and students.

A correction has been made to the **Materials and Methods**, **Participants and Procedures**, Paragraph One:

“This study was conducted with a convenience sample from a mixed age class of 4, 5, and 6-year-old students in an early childhood classroom (*N* = 25). This classroom is part of a larger early childhood development center that is facilitated and associated with a small, public university in the southeast of the United States. The student population represents families associated with the college or local community. As a demonstration program, the school structures class groups to support appropriate diversity that reflects the surrounding community, gender distribution, and accommodation for children with special needs to model best practices for pre-service teachers.”

A correction has also been made to the **Results**, **Stage Two Themes**, in the following sections:

**Stage 1: Pre-Storytelling**, paragraph three:

“Lewis, a 4-year-old male student wrote, “A bad lightning storm,” (picture 1, 10/16/17), “Bad lightning storm, good lightning storm,” (pictures 2 & 3), and “The big lightning storm,” (picture 4). See **Figure 1** for pictures 1–4. This student often draws an image first, then will say a similar story several days in a row or will revert to the same story frequently. A recent hurricane made landfall in the local area and this impacted the lives of all students, which became a common thread in many student stories that semester. Students in Lewis's group also wrote about their homes being flooded or having to leave and stay with relatives during the weeks that followed. Researcher notes indicate that Lewis preferred to write about the storm when other ideas did not present themselves. “When he doesn't want to write he'll write about a storm.””

**Stage 2: Developing Storyteller**, paragraph three:

“Norman, a 4-year-old male student, is obsessed with Transformers. His stories all reflect his knowledge and understanding of either a particular movie or show. Each story has the same characters playing their designated roles, Optimus Prime is the good guy, Megatron the bad guy. These characters fight and the good guys always win. Researcher notes capture a conversation Norman had with a teaching assistant as he creates and draws his story.”

**Stage 3: Emerging Storyteller**, paragraph three:

“A 6-year-old male student, Orlando enjoys playing with Legos, Mixels, and is familiar with superhero stories. His stories reflect a combination of a variety of characters interacting in new and creative ways. He has moved away from a script and can write both fiction and personal narratives, expressing what is familiar to him: “Antman tricked the bad guy to sit on the electric computers and then Antman goes to a secret tunnel that goes to the batcave and Superman's hideout” (picture 8). On other days this student can recount a personal event: “I built things with my legos. You click them apart and make funny things” (picture 9). He is able to tell the difference between real and make believe. See **Figure 3** for pictures 8 and 9.”

**Stage 4: Early Storyteller**, paragraph three:

“Sally, a 5-year-old female student, usually writes in coordination with several of her friends. They write on the same themes and consult with the teachers as they draw. An example of this would be a question Sally asks as she draws a version of Thomas, another student, “What color shirt do you want me to draw for you?” In another example, Sally told the classroom teacher “I'm going to write about my grandparents.” She had a plan for her story when she arrived at school, and immediately sat down to draw and write when she entered the classroom. She crafted her story with some input from Rose, a 6-years-old peer, to help her spell rainbow and parents. She reviewed her story by reading, “I saw a rainbow with my grandparents then I went to school to play on the seesaw with Jiiel” to her tablemates (picture 11). See **Figure 5** for picture 11.”

Finally, the Acknowledgements statement was also omitted it has now been added: “Jamie Fecio and Kailey Ray were graduate assistants at the early childhood development center and assisted in instruction and data collection in the study.”

The authors apologize for these errors and state that they do not change the scientific conclusions of the article in any way. The original article has been updated.

## Conflict of Interest Statement

The authors declare that the research was conducted in the absence of any commercial or financial relationships that could be construed as a potential conflict of interest.

